# SNEP: Simultaneous detection of nucleotide and expression polymorphisms using Affymetrix GeneChip

**DOI:** 10.1186/1471-2105-10-131

**Published:** 2009-05-06

**Authors:** Hironori Fujisawa, Youko Horiuchi, Yoshiaki Harushima, Toyoyuki Takada, Shinto Eguchi, Takako Mochizuki, Takayuki Sakaguchi, Toshihiko Shiroishi, Nori Kurata

**Affiliations:** 1 The Institute of Statistical Mathematics, Tokyo 106-8569, Japan; 2 Plant Genetics Laboratory, National Institute of Genetics, Mishima, Shizuoka 411-8540, Japan; 3 Mammalian Genetics Laboratory, National Institute of Genetics, Mishima, Shizuoka 411-8540, Japan; 4 Transdisciplinary Research Integration Center, Research Organization of Information and Systems, Tokyo 105-0001, Japan

## Abstract

**Background:**

High-density short oligonucleotide microarrays are useful tools for studying biodiversity, because they can be used to investigate both nucleotide and expression polymorphisms. However, when different strains (or species) produce different signal intensities after mRNA hybridization, it is not easy to determine whether the signal intensities were affected by nucleotide or expression polymorphisms. To overcome this difficulty, nucleotide and expression polymorphisms are currently examined separately.

**Results:**

We have developed SNEP, a new method that allows simultaneous detection of both nucleotide and expression polymorphisms. SNEP involves a robust statistical procedure based on the idea that a nucleotide polymorphism observed at the probe level can be regarded as an outlier, because the nucleotide polymorphism can reduce the hybridization signal intensity. To investigate the performance of SNEP, we used three species: barley, rice and mice. In addition to the publicly available barley data, we obtained new rice and mouse data from the strains with available genome sequences. The sensitivity and false positive rate of nucleotide polymorphism detection were estimated based on the sequence information. The robustness of expression polymorphism detection against nucleotide polymorphisms was also investigated.

**Conclusion:**

SNEP performed well regardless of the genome size and showed a better performance for nucleotide polymorphism detection, when compared with other previously proposed methods. The R-software 'SNEP' is available at .

## Background

Affymetrix GeneChip expression arrays are high-density short oligonucleotide microarrays that were initially designed to monitor genome-wide expression profiles [1]. Affymetrix probe sets consist of several (typically 11) 25-mer short oligomer probes matching each gene [perfect match (PM) probes] and accompanying probes with single complementary substitutions in the 13th base of each PM probe [mismatch (MM) probes]. Signal intensities for the probes are obtained by hybridizing labeled genomic DNA (gDNA) or mRNA to the expression array. Recently, nucleotide polymorphisms have been detected with these probes by hybridizing gDNA from human malaria parasite [2], yeast [3], malaria mosquito [4], *Arabidopsis *[5,6], and rice [7], and by hybridizing mRNA from yeast [8], *Arabidopsis *[9], barley [10-12], maize [13], and mammals [14]. A nucleotide polymorphism observed at a probe level was called a single feature polymorphism (SFP) by Borevitz *et al. *[5]. An expression polymorphism was defined as a difference in gene expression levels between strains (or species), which can be used as a gene expression marker [9]. Because an expression array can be used for detecting both expression and nucleotide polymorphisms, the expression array has the potential to be a powerful tool for identifying functional variants that are associated with morphological, physiological, and/or ecological diversity within and between strains (or species).

In contrast, when different strains (or species) produce different signal intensities after mRNA hybridization, it is not easy to determine whether the signal intensities are affected by nucleotide or expression polymorphisms. Thus, it has been noted that caution should be used when evaluating gene expression levels in cross-strain (or cross-species) hybridization using expression arrays [13,15,16]. To overcome this difficulty, nucleotide and expression polymorphisms are currently examined separately. In this paper, we simultaneously examine these two types of polymorphism to effectively detect them.

Ideally, we assume that the signal intensity ratios for two strains are almost the same on all the probes when no SFP probes are present in a probe set. This was similarly adopted in Ronald *et al. *[8], Cui *et al. *[11] and Luo *et al. *[12]. Here, we suppose that this assumption may not hold for SFP probes because a nucleotide polymorphism can reduce hybridization signal intensity. Therefore, the signal intensities from the SFP probes may be regarded as outliers. Based on these premises, we have constructed a statistical model and then detected nucleotide and expression polymorphisms using a robust procedure against the outliers. The proposed method is referred to as 'Simultaneous detection of Nucleotide and Expression Polymorphisms (SNEP)'.

SFPs between two strains are easily detected when gDNA hybridization is feasible, because the amounts of applied target DNA are thought to be almost the same between the two strains for each probe. This allows us to easily detect SFPs, e.g., by a simple *t*-test for each probe. However, gDNA hybridization can only be used with smaller genomes. For larger genomes, such as barley and mammals, mRNA hybridization should be used instead, because the significant cross hybridization is observed during whole genome hybridization [17,18]. When mRNA hybridization is employed, the amount of applied target cRNA for a given gene is not always the same between the two strains, which makes simple *t*-tests infeasible; therefore some methods for detecting SFP probes have been developed. Rostoks *et al. *[10] adopted a standard testing procedure based on a standard interaction model with significance analysis of microarrays, SAM [19], and detected SFP probes by a significant interaction of probe by genotype. Similar to our study, Cui *et al. *[11] regarded the signal intensities from the SFP probes as outliers and adopted a robust projection pursuit to detect the SFP probes. These two groups used their methods to examine barley. Ronald *et al. *[8] focused on the ratio of signal intensity to the gene expression level for each probe; if the ratio was different between two genomes, then the probe was judged to be an SFP. They applied their method to *S. cerevisiae*. Luo *et al. *[12] used a similar strategy of analyzing the signal intensity ratio. Greenhall *et al. *[14] compensated for gene expression differences by appropriately scaling the PM minus MM values and detected the SFP probes by a simple *t*-test.

## Methods

### Model and hypothesis

**Figure 1:**
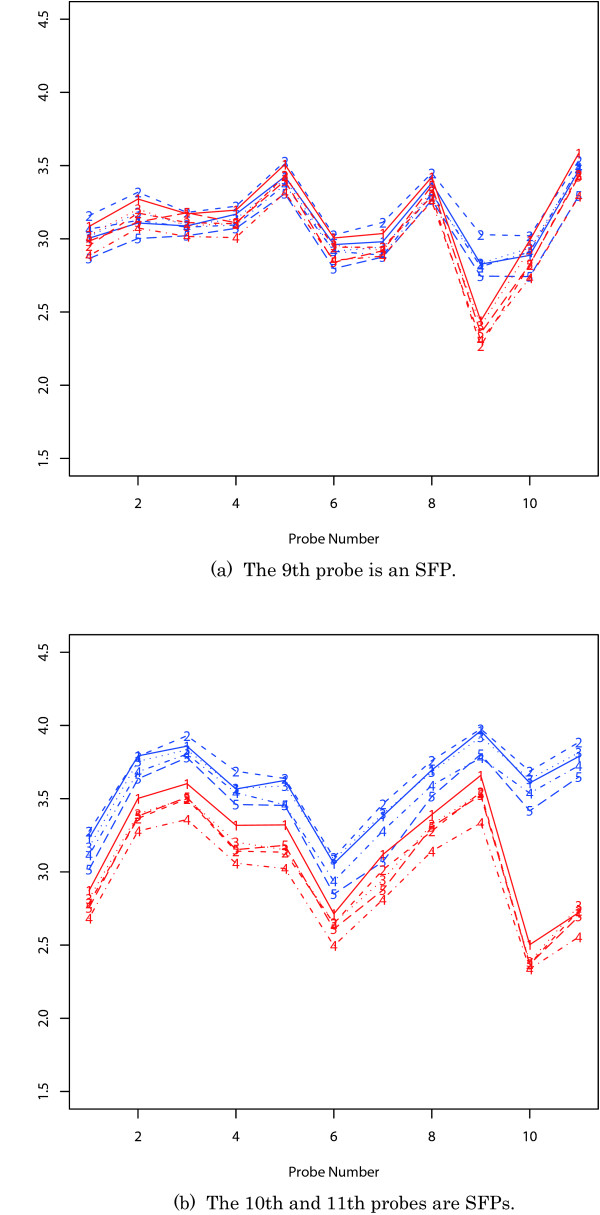
**mRNA data by GeneChip**. Each figure contains data for a different gene. The *x*-axis indicates the 11 probes. The *y*-axis is the log10 value of signal intensity. The blue and red lines correspond to two strains. The line number indicates the replicate number.

Figure 1 shows two typical sets of mRNA data. Based on these data, we have constructed a basic statistical model and next prepared some hypotheses. Hereafter, the log10 value of signal intensity is called the 'log-intensity' for simplicity.

Let *x*_*ijk *_be the log-intensity in the *k*th replicate on the *j*th probe for the *i*th strain, where *i *= 1, 2 stand for two strains. Let *μ*_*ij *_be the mean log-intensity on the *j*th probe for the *i*th strain. Here we assume that the difference between the log-intensity and the mean does not depend on the position of the probe. This tendency was seen for many probe sets as well as in Figure 1 and similarly adopted in [8,11,12]. To express this characteristic difference, we use the parameter *ν*_*ik *_in the *k*th replicate for the *i*th strain. Consequently, the basic statistical model can be expressed as



where , *ε*_*ijk*_'s are the noise terms, *J *is the number of probes and *K *is the number of replicates. We assume that *ε*_*ijk *_has a normal distribution with mean zero and variance *σ*^2^.

If no SFP is present in a probe set, then the log-intensity differences between two strains are expected to be almost the same on all the probes in the probe set, in other words, the signal intensity ratios for two strains are almost the same on all the probes in the probe set. This tendency was seen for many probe sets as well as in Figure 1 with the exception of some SFP probes. Let the difference between two means be denoted by *λ*_*j *_= *μ*_1*j *_- *μ*_2*j*_. The above expectation implies the hypothesis that



In this paper, the difference between *λ*_*j *_and the common *λ *is called the 'probe effect'.

In Figure 1, some *λ*_*j*_'s are clearly larger than the common *λ*. This would be caused by SFPs, because the nucleotide polymorphism can reduce hybridization signal intensity. The alternative hypothesis can be expressed as a one-sided one, given by



which means that *λ*_*j *_is larger (or smaller) than the common *λ *and the *λ*_*j*' _'s except for the *j*th probe are the same as the common *λ*. If we reject the null hypothesis *H *and accept the alternative hypothesis *K*_*j*_, then we will judge the *j*th probe to be an SFP for the second (or first) strain. For the 9th probe in Figure 1(a) and the 10th and 11th probes in Figure 1(b), we expect to accept the alternative hypothesis *K*_*j*_: *λ*_*j *_> *λ*.

Consider the case where the first strain is the platform one, in other words, the hybridization can be disturbed only for the second strain. Then we use only one alternative hypothesis, such as *K*_*j*_: *λ*_*j *_> *λ*, because *μ*_2*j *_can become much smaller than expected.

Let us prepare the hypothesis *H*_0_: *λ *= 0. If the null hypothesis *H*_0 _is rejected, then the corresponding gene is judged to be differently expressed. For Figures 1(a) and 1(b), we expect to accept and reject the null hypothesis *H*_0_: *λ *= 0, respectively.

### Random effects

We also incorporate random effects into the model to address various types of dispersion, including the noise dispersion. Such a device is often adopted to reduce the number of parameters when the number of replicates is small. This device also makes the robust procedure easily applicable, as described later. We assume that the difference in the means, *λ*_*j *_= *μ*_1*j *_- *μ*_2*j*_, has a normal distribution with mean *λ *and variance *τ*^2 ^because *λ*_*j*_'s can be regarded to be dispersed around the common difference *λ*.

### Treatment of outlier

The following is a simple review about the adverse effects of an outlier. Let *y*_1_,..., *y*_10 _be the observations. Let *y*_1 _= ⋯ = *y*_9 _= 0 and *y*_10 _= 50. Consider the estimation of the mean parameter *μ *= E [*y*]. The sample mean, a standard estimate of the mean parameter, is 5. This estimate may be inappropriate because we generally regard *y*_10 _= 50 as an outlier and expect *μ *= 0 from the other observations. To carefully treat outliers, we often adopt the robust parameter estimation.

The signal intensities from the SFP probes can be regarded as outliers when they show different behaviors from other signal intensities, as seen in Figure 1. Thus we need to carefully examine the RNA data and then adopt a robust procedure against the outliers, as described later.

**Figure 2:**
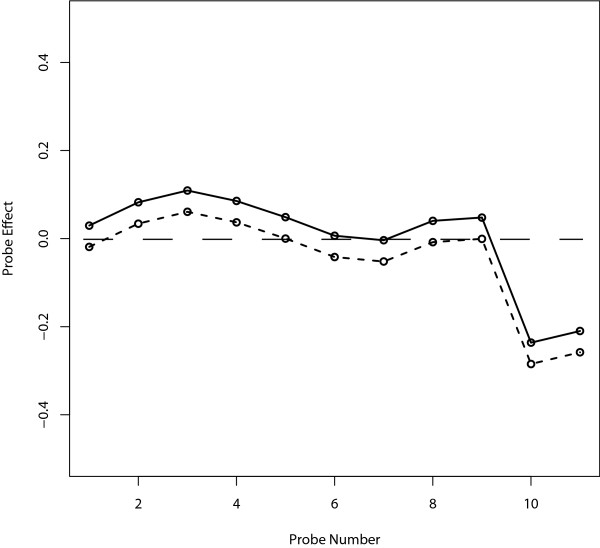
**Estimate of the probe effect (*λ*_*j *_- *λ*)**. The *x*-axis indicates the 11 probes. The *y*-axis denotes the estimate of the probe effect from Figure 1(b). The dashed and solid lines correspond to the robust estimate and the standard estimate (maximum likelihood estimate), respectively.

The parameter estimation of the probe effect (*λ*_*j *_- *λ*) is illustrated in Figure 2. The probe effect is expected to be close to zero if the probe is not an SFP. Except for the 10th and 11th SFP probes in Figure 1(b), the robust estimates were balanced on both sides of zero, whereas most of the standard estimates (maximum likelihood estimates) were greater than zero.

### Parameter estimation

Let  and . Let . Here we assume that the hypothesis *H *holds. Then, , where *κ*^2 ^= *τ*^2 ^+ 2*σ*^2^/*K*. Let us consider the parameter estimation based on *z*_*j*_'s.

Let ɸ(*z*; ***θ***) be the normal density function with the parameter ***θ ***= (*λ*, *κ*^2^). The robust parameter estimate  can be obtained by the minimizer of



This type of robust parameter estimation was investigated by Windham [20], Basu *et al. *[21], Jones *et al. *[22], and Fujisawa and Eguchi [23].

The positive tuning parameter *γ *controls the trade-off between efficiency and robustness. As *γ *goes to zero, the  limits to the maximum likelihood estimator, which is efficient but not robust against outliers. When *γ *= 1, the  is similar to the *L*_2 _estimator, which is known as a strong robust estimator [24]. As the tuning parameter *γ *is smaller or larger, the robustness will become weaker or stronger, respectively, whereas the efficiency will increase or decrease, respectively. We used *γ *= 0.5 for the analysis of the mRNA data from various experiences. We will also discuss the choice of *γ *later.

The normal density function *ɸ*(*z*; ***θ***) belongs to an exponential family. For this reason, we can construct a convenient and iterative algorithm to obtain the robust parameter estimate (Appendix A1 of the additional file 1). By virtue of the standard theory of M-estimation, the distribution of the robust parameter estimator can be approximated to a normal distribution (Appendix A2 of additional file 1). The above robust parameter estimation shows strong robustness even when the ratio of the outlier is not small. This is suitable for the analysis of the mRNA data because the probe set may contain a number of SFP probes. For the detailed properties of the above robust parameter estimation, see Fujisawa and Eguchi [23].

It should be noted that the variance parameter *κ*^2 ^= *τ*^2 ^+ 2*σ*^2^/*K *includes two types of variance parameter, *τ*^2 ^and *σ*^2^. The estimate  is sometimes underestimated for the analysis of the mRNA data when *σ*^2 ^is relatively large and the number of replicates is small. To overcome this difficulty, we modified the estimate  as follows. We first estimated the variance parameter *σ*^2 ^by a standard unbiased estimate  and then replaced the estimate  by  if , because *κ*^2 ^≥ 2*σ*^2^/*K*.

### Testing procedure

Consider the testing problem for the null hypothesis *H*: *λ*_1 _= ⋯ = *λ*_*J *_= *λ *against the alternative hypothesis *K*_*j*_: *λ*_*j *_> *λ*. If we can make an appropriate estimate  of *λ*_*j*_, then we can propose the Wald-type test statistic:



where  is an appropriate estimate of the standard deviation of  (Appendix A3 of additional file 1). We simply estimated the parameter *λ*_*j *_by . The distribution of the test statistic *T*_*j *_can be approximated to the standard normal distribution. Let *z*_*α *_be the upper 100α % point of the standard normal distribution. If *T*_*j *_> *z*_*α*_, then we will accept the alternative hypothesis *K*_*j *_at significance level α and judge the *j*th probe as an SFP. By a similar way, we can also treat the alternative hypothesis *K*_*j*_: *λ*_*j *_<*λ *and two-sided alternatives.

Consider the testing problem for the null hypothesis *H*_0_: *λ *= 0. We can propose the Wald-type test statistic:



where  is an appropriate estimate of the standard deviation of  (Appendix A4 of the additional file 1). If |*T*_0_| > *z*_*α*/2_, then we will reject the null hypothesis *H*_0 _at significance level *α *and judge the corresponding gene to be differently expressed.

### mRNA data

The Affymetrix GeneChip Rice Genome Array consisted of 57,381 probe sets containing 631,066 probes. Signal intensities of two fully sequenced rice cultivars,  *japonica *rice "Nipponbare" [25] and *indica *one "93-11" [26], were observed by hybridizing their mRNA to the rice array. mRNA data were obtained for five biological replicates from 2 cm young panicles of both Nipponbare and 93-11.

The Affymetrix GeneChip Mouse Genome 430 2.0 Array consisted of 45,101 probe sets containing 496,468 probes. Signal intensities of two inbred strains, C57BL/6J (referred to below as B6) and MSM/Ms (*Mus musculus molossinus*), were observed by hybridizing their mRNA to the mouse array. mRNA data were obtained for two biological replicates from the liver of both B6 and MSM/Ms.

For a more detailed experimental environment and sequence analysis, see the additional file 1. All microarray data from this study are available from the Center for Information Biology gene EXpression (CIBEX) database  under accession numbers CBX50 and CBX54.

## Results

### SFP detection in barley data

The barley data were analyzed by Rostoks *et al. *[10]. To detect SFP probes, they adopted a standard testing procedure based on a standard interaction model with SAM [19]. There were 2,601 probes whose target sequences were confirmed in the two analyzed varieties: Morex and Golden Promise. They consisted of 2,200 non-polymorphic probes and 401 polymorphic probes among which 178 and 223 probes were polymorphic to Morex and Golden Promise sequences, respectively. There were six types of tissue and all except one were analyzed using three replicates ().

We considered both alternative hypotheses, *K*_*j*_: *λ*_*j *_> *λ *and *K*_*j*_: *λ*_*j *_<*λ*, because a probe could be an SFP for both strains. The sensitivity was calculated by the ratio of the number of probes correctly judged as SFPs to the number of SFP probes. The false positive rate (FPR) was calculated by the ratio of the number of probes incorrectly judged as SFPs to the number of probes judged as SFPs. The sensitivities and FPRs of various methods are given in Table 1.

**Table 1:** Sensitivity and FPR of SFP detection in barley data.

Tissue	COL			
Method	SNEP	LRT	Ros^*a*^	Gre^*b*^
Sensitivity	0.579	0.569	0.52	0.506
FPR	0.259	0.299	0.35	0.420
Tissue	CRO			
Method	SNEP	LRT	Ros^*a*^	Gre^*b*^
Sensitivity	0.673	0.636	0.58	0.608
FPR	0.173	0.338	0.34	0.440

Tissue	GEM			
Method	SNEP	LRT	Ros^*a*^	Gre^*b*^
Sensitivity	0.691	0.618	0.63	0.534
FPR	0.153	0.218	0.34	0.314

Tissue	LEA			
Method	SNEP	LRT	Ros^*a*^	Gre^*b*^
Sensitivity	0.574	0.531	0.51	0.524
FPR	0.151	0.273	0.34	0.440

Tissue	RAD			
Method	SNEP	LRT	Ros^*a*^	Gre^*b*^
Sensitivity	0.656	0.623	0.62	0.504
FPR	0.137	0.264	0.34	0.276

The barley data were obtained from the supplementary data at [10]. COL: coleoptile. CRO: seedling crown. GEM: embryo from germinating seed. LEA: seedling leaf. RAD: radicle (seminal root). The number of replicates was three (*K *= 3) for each tissue.

Ros^*a*^: Method of Rostoks *et al. *[10]. The corresponding sensitivity and FPR were extracted from Rostoks *et al. *[10].

Gre^*b*^: Method of Greenhall *et al. *[14]. Approximately 10% probes of 2,601 candidate probes were not used when calculating the sensitivity and FPR, because these probes did not meet the authors' selection criteria.

SNEP was applied to the barley data with three replicates (*K *= 3) without normalization. The significance levels were set at 10^-3^/2 and 10^-2^/2 for the first four and RAD samples, respectively, which allowed us to easily compare SNEP with the method employed by Rostoks *et al. *[10]. SNEP markedly outperformed their method. For example, for the CRO samples, the sensitivity and FPR of SNEP were approximately 9% and 17% superior to those obtained using their method, respectively.

We also applied the likelihood ratio test (LRT) based on a standard interaction model without normalization. LRT is a standard testing procedure that is similar to the basic test statistic used by Rostoks *et al. *[10]. The significance levels for LRT were set at 10^-5^/2 and 10^-3^/2 for the first four and RAD samples, respectively, which allowed us to easily compare LRT with the other methods. SNEP markedly outperformed LRT, whereas LRT almost outperformed the methods of Rostoks *et al. *[10].

We also analyzed the same barley data with the method employed by Greenhall *et al. *[14]. Both of the one-sided alternative hypotheses were considered and the significance level was set at 10^-4^/2 by using the standard normal approximation. Both SNEP and LRT markedly outperformed their method.

The method of Rostoks *et al. *[10] and LRT were based on a similar standard testing procedure, but the former was inferior to the latter. The major difference between two methods was that the former adopted the normalization and SAM. It might seem that the normalization markedly affected the performance of the method. A prerequisite for most normalization is that the mRNA affinities to the microarray are the same for all of the replicates. Normalization enables the total amount of hybridized mRNA to be equalized for all of the replicates. However, in different strains (or species), nucleotide polymorphisms affect the mRNA affinities to the microarray. For cross-strain (or cross-species) microarrays, normalization is not sufficient to equalize the affinities. Thus, we did not include normalization in SNEP.

**Figure 3:**
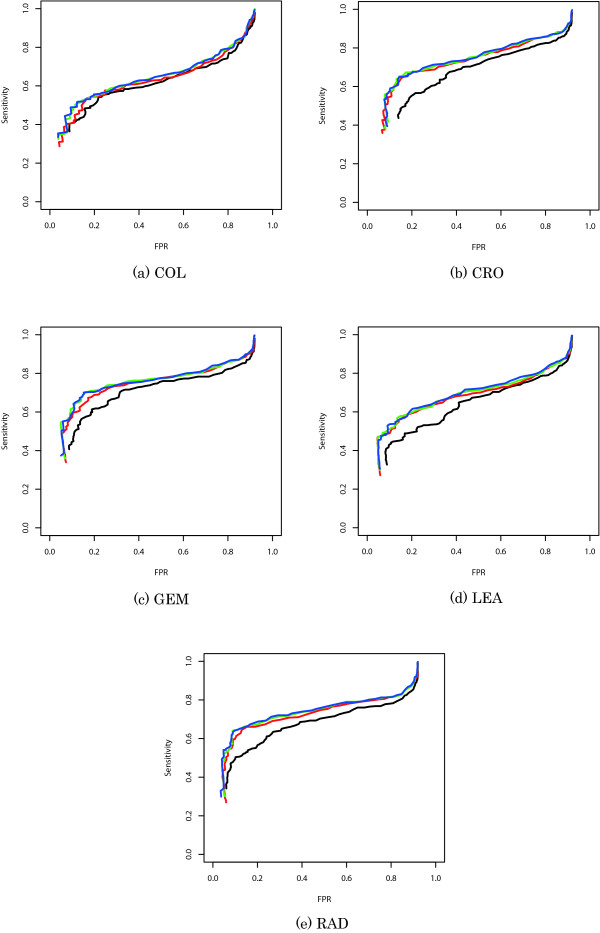
**ROC curve for SFP detection**. The *x*- and *y*-axes are the FPR and sensitivity, respectively. The black, red, green and blue lines are based on LRT, SNEP(*γ *= 0.2), SNEP(*γ *= 0.5) and SNEP(*γ *= 0.8), respectively, using appropriate significance levels, which range from 10^-16 ^to 1.

Receiver operating characteristic (ROC) curves are shown in Figure 3. One method is said to outperform another when the associated ROC curve lies above the ROC curve of the comparator method. SNEP markedly outperformed LRT. In particular, when the FPR was approximately 0.2, the difference between the results obtained by SNEP and LRT tended to become larger as the FPR became smaller. We also tried to use *γ *= 0.2 and *γ *= 0.8 instead of *γ *= 0.5, which was the default value for SNEP. The results obtained with *γ *= 0.2 was worse than those obtained with the other values. It was also pointed out in Fujisawa and Eguchi [23] that the case *γ *= 0.2 would not suffice when the ratio of outlier was not small. We could not clearly determine which was better, *γ *= 0.5 or *γ *= 0.8. Because the objective function for robust parameter estimation tends to be flat for a large value of *γ*, the iterative algorithm for robust parameter estimation did not work well for synthetic data sets with a large value of *γ *(data not shown). Therefore, we used *γ *= 0.5 as the default value for SNEP.

### SFP detection in rice data

SNEP was also applied to the rice data. Two points made this analysis different from that performed with the barley data. For barley, the genome sequence was only partly known, whereas we obtained the whole genome sequences of both Nipponbare and 93-11. We thus could study the performance of the methods in more detail. The rice arrays were designed mostly with *japonica *transcripts and therefore Nipponbare was regarded as the platform strain.

We prepared 'canonical rice data' and then we applied SNEP and LRT to the canonical rice data. The canonical rice data consisted of signal intensities for the probe sets in which all 11 probe sequences were perfectly matched as a single copy in the Nipponbare genome and were matched as a single copy in the 93-11 genome. Note that the term 'single copy' means there is only one similar sequence in a genome. For the canonical rice data, the SFP probes only interacted with the 93-11 sequences. For this reason, we used only one alternative hypothesis to detect SFP probes for 93-11.

We first examined the effects of the degree of signal intensity by the median of the log-intensities in a probe set, called the 'median-intensity', because we thought that low signal intensity level might not represent enough hybridization. SNEP was applied to the canonical rice data at significance level 10^-3^. We constructed four classes (<2, 2–2.5, 2.5–3, and 3≤) of the median-intensity and categorized the probe sets into each class. The sensitivity and FPR of SNEP were calculated for each class when the number of sequence-verified SFP probes in a probe set, called the 'SFP number', was one (Table 2). It was clear that the sensitivity and FPR were much worse when the median-intensity was low. Moreover, when the median-intensities for both strains were more than 2.5, the sensitivity and FPR were stable at high and low levels, respectively. Thus, we say that the gene is sufficiently expressed when the median-intensity is more than 2.5.

**Table 2:** Sensitivity and FPR of SFP detection for various signal intensities in the canonical rice data in which the SFP number was one.

	< 2	2 – 2.5	2.5 – 3	3 ≤
	# of probe sets			
< 2	186	6	0	0
2 – 2.5	322	1184	18	1
2.5 – 3	6	136	452	9
3 ≤	0	5	115	422

	# of SFP probes judged by SNEP			
< 2	10	3	0	0
2 – 2.5	70	338	24	0
2.5 – 3	8	99	415	9
3 ≤	0	5	111	415

	Sensitivity			
< 2	0.022	0.333	NA	NA
2 – 2.5	0.034	0.164	0.722	0.000
2.5 – 3	0.167	0.529	0.750	0.667
3 ≤	NA	0.200	0.843	0.777
	FPR			
< 2	0.600	0.333	NA	NA
2 – 2.5	0.843	0.426	0.458	NA
2.5 – 3	0.875	0.273	0.183	0.333
3 ≤	NA	0.800	0.126	0.210

. : median-intensities for Nipponbare and 93-11. NA: not available.

SNEP and LRT were applied to the canonical rice data in which the genes were sufficiently expressed. The significance levels for SNEP and LRT were set at 10^-3 ^and 10^-4^, respectively, which allowed us to easily compare the two methods. The sensitivity and FPR are given in Table 3. We omitted the extreme case in which all 11 probes were SFPs, because there were much more extreme cases compared with the other cases. The sensitivity and FPR became smaller as the SFP number became larger, as expected. In contrast to the analysis of the barley data, SNEP only slightly outperformed LRT. This would be because the disadvantages associated with LRT are not so remarkable in general when using only one of the two one-sided alternative hypotheses.

**Table 3:** Sensitivity and FPR of SFP detection for various SFP numbers in the canonical rice data in which the genes were sufficiently expressed.

SFP number	1	1–3	1–5	1–10
# of probe sets	998	1699	1831	1901
# of SFP probes	998	2602	3177	3689
	SNEP			
Sensitivity	0.772	0.747	0.717	0.653
FPR	0.189	0.112	0.101	0.097
	LRT			
Sensitivity	0.800	0.748	0.711	0.666
FPR	0.222	0.132	0.119	0.113

We also examined an alternative way of selecting genes. We used Affymetrix GeneChip Operating Software (GCOS), which has been often used to determine whether or not a probe is sufficiently expressed. We calculated the sensitivity and FPR of SNEP for detecting SFP probes in the probe sets in which all 11 probes were judged to be 'Present' at significance level 0.01(default) by GCOS. When the SFP number was one, the sensitivity and FPR became approximately 13% and 3% worse than those shown in Table 3.

### SFP detection in mouse data

SNEP was also applied to the mouse data. The mouse genome might be more complex than the rice genome, because the mouse genome is roughly six times larger, but mice contain less than half the number of genes identified in rice [25-27]. Because the mouse array was designed for B6 transcripts, we used only one alternative hypothesis to detect SFP probes for MSM/Ms. The overall nucleotide substitution rate between these two strains was as high as 0.0096 [28]. The genome sequence of MSM/Ms has been extensively studied and 187,560 probe target sequences from MSM/Ms were available at . We used 17,043 probe sets in which all 11 probe target sequences were known in both B6 and MSM/Ms.

SNEP was applied to the mouse data in which the median-intensity was more than 2.5 and the SFP number was one. There were 710 objective probe sets. The significance level was set at 10^-3^. The sensitivity and FPR of SNEP were 0.524 and 0.316, respectively, which were inferior to the values obtained in a similar analysis of rice data (0.772 and 0.189). However, because the mouse genome is more complicated, it seemed that the performance of SNEP was still good.

### Effects of signal intensity level for SFP detection

In the analysis of mouse data, we increased the threshold value from 2.5 to 3 in order to avoid the effects of cross-hybridization. There were 165 objective probe sets. The sensitivity and FPR were improved to 0.733 and 0.243, respectively. We also used the threshold value 2.5 to examine the barley data. We first focus on the analysis of the COL samples. The number of objective probes was reduced from 2,601 to 1,937. The sensitivity and FPR did not markedly change in comparison to those obtained in the analysis of the rice data. We further increased the threshold value from 2.5 to 3. The number of objective probes was reduced to 838. The sensitivity and FPR were much improved from 0.579 and 0.259 to 0.810 and 0.156, respectively. For other types of tissue, similar improvements were observed. Thus, the way of selecting genes will be an important issue to stabilize the SFP detection.

### Detecting differently expressed genes in rice data

In contrast to SFP detection, it is difficult to clearly investigate the performance of the method for detecting differently expressed genes, due to the paucity of data regarding which genes are differently expressed. Instead, we examined whether SNEP was robust against the adverse effects of an SFP probe for detecting differently expressed genes. We compared the robust test statistic *T*_0 _with the standard *t*-statistic based on the Tukey's biweight estimate. We adopted the Tukey's biweight estimate instead of directly using the raw data, because this estimate has been commonly used for detecting differently expressed genes.

We first illustrate the robustness of the two test statistics against the adverse effects of an SFP probe by analyzing the data in Figure 1(a), which suggests the hypothesis that the gene is not differently expressed. The *T*_0_-value was 1.53 and the *p*-value was 0.126. This result was consistent with our hypothesis. However, the *t*-value was 3.64 and the *p*-value was less than 10^-3^. This result was not consistent with our hypothesis. The Tukey's biweight method tends to weaken the adverse effects of an outlier, but it is not always designed to weaken the adverse effects of an SFP probe because it is based on only one strain. In fact, the signal intensities on the 9th probe may not be outliers when we focus only on each replicate for 93-11 and neglect the other replicates. In such a case, the Tukey's biweight method produces a smaller estimate of gene expression level for 93-11, which results in a larger *t*-statistic. These would be the reason why the *t*-value was larger than expected. SNEP can weaken the adverse effects of an SFP probe because it examines two strains simultaneously, as described already.

**Figure 4:**
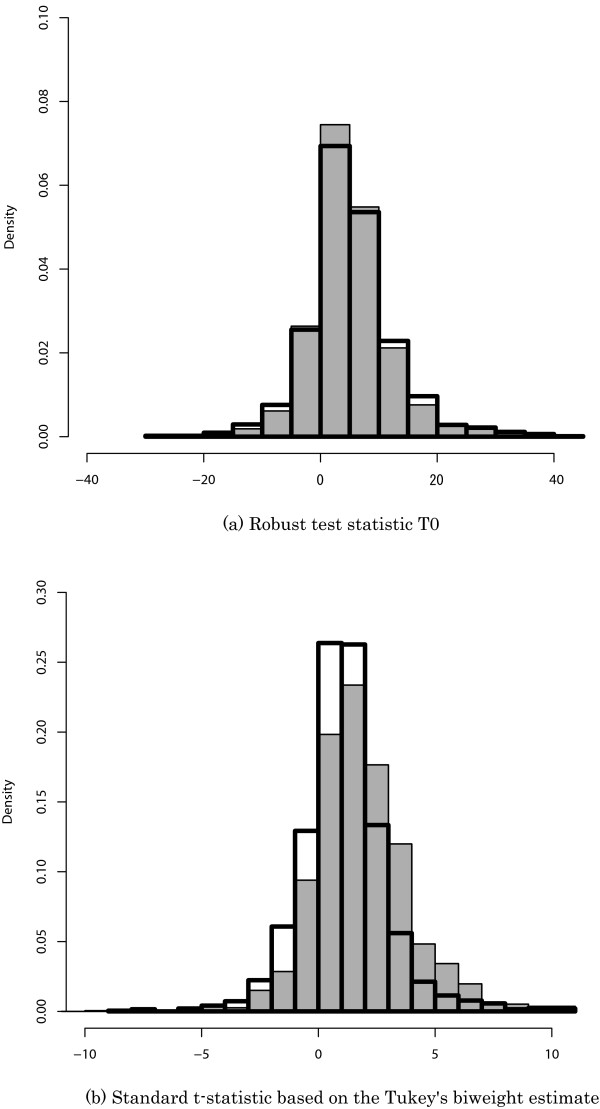
**Distribution of the test statistics**. The gray histograms are based on the canonical rice data in which the genes were sufficiently expressed and the SFP number ranged from 1 to 5. The unshaded histograms are based on the modified data in which the signal intensities from the SFP probes were deleted.

Figure 4 shows the global robustness of the two test statistics by comparing two cases. One case was based on the canonical rice data in which the genes were sufficiently expressed and the SFP number ranged from 1 to 5. The other case was based on the modified data in which the signal intensities from the SFP probes were deleted. The former case might be affected by the SFP probes, because the data included the signal intensities from the SFP probes. If an SFP probe produced an adverse effect, the test statistic would tend to be larger in the former case than in the latter case, as illustrated above. As seen in Figure 4, the *t*-statistic tended to be larger in the former case, whereas the *T*_0_-statistic did not. We showed that the robust test statistic *T*_0 _was much more robust against the adverse effects of an SFP probe than the *t*-statistic based on Tukey's biweight estimate. We also found that the absolute value of the *T*_0_-statistic tended to be slightly smaller in the former case than in the latter case. This may occur because the signal intensities from the SFP probes are not always outliers. In such a case, the sample size of meaningful probes tends to become large and then the denominator of *T*_0 _tends to become small.

## Conclusion and discussion

We have developed 'SNEP' to simultaneously detect nucleotide and expression polymorphisms. We expected that the signal intensity ratios for two strains were almost the same on all the probes in a probe set when no SFPs were present. We furthermore considered that the SFP probe could be regarded as an outlier because the SFP probe might not satisfy this expectation. To effectively use these ideas, we adopted a statistical model and a robust procedure.

SNEP was applied to data from barley (large genome that has not been extensively sequenced), rice (small genome that has been extensively sequenced) and mice (large genome that has been extensively sequenced). When a great deal of sequence information was available, one of the two strains (or varieties) was regarded as a platform strain. SNEP worked well regardless of genome size. SNEP outperformed the standard testing procedure, the method of Rostoks *et al. *[10] and the method of Greenhall *et al. *[14] for detecting SFP probes in the barley data. SNEP also performed well for detecting SFP probes in the rice and mouse data. SNEP was more powerful for detecting SFP probes than the standard testing procedure when no platform strain was present, in other words, when both alternative hypotheses were necessary. SNEP was carried out without normalization and the effect of normalization was also investigated. SNEP was more robust against the adverse effect of an SFP probe for detecting differently expressed genes than the standard *t*-statistic based on the Tukey's biweight estimates.

It is worth noting that there may be more than two SFP probes in a given probe set, which typically consisted of 11 probes. In this case, the ratio of the outlier is not small generally, making it difficult to appropriately obtain statistical results. To overcome this difficulty, SNEP uses a divergence-based procedure. This procedure is robust even in the cases where the ratio of the outlier is not small, and provides some convenient properties. Cui *et al. *[11] also adopted a robust procedure based on projection pursuit using the median, but the projection pursuit was computationally heavy and furthermore the median might suffer from a heavy bias because the median had no redescending weight [29].

Differently expressed genes can be detected by a simple *t*-test based on estimated gene expression levels. The gene expression levels are typically estimated by the Tukey's biweight method. This method tends to weaken the adverse effects of an outlier, but it is not always designed to weaken the adverse effects of an SFP probe because it is based on only one strain. However, SNEP can weaken the adverse effects of an SFP probe because it addresses two strains simultaneously. Some studies showed that SNEP was more robust against the adverse effects of an SFP probe for detecting differently expressed genes than a simple *t*-test based on the Tukey's biweight estimates.

New DNA sequencing methods, which can produce hundreds of millions of DNA sequence reads during a single run, are superior to microarray technology in both sequence variation detection and gene expression level estimation [30,31]. However, the cost of a single run using a "next generation sequencer" is still four to five times higher than that with Affymetrix GeneChip. Array technologies are a cost-effective option for studies of biodiversity. SNEP offers a reliable tool for detection of both nucleotide sequence and expression level variations in small or large genomes during array analysis.

## Authors' contributions

HF proposed the idea of the method, performed the statistical analysis, made the software, and drafted the manuscript. YH performed the rice experiments and statistical analysis. YH discussed the rice experimental design, analyzed the rice probe sequences, and helped to draft the manuscript. TT performed the mouse experiments and helped to draft the manuscript. SE improved the method. TM analyzed the rice probe sequences. TS helped to perform the statistical analysis and make the software. TS conceived of the study and participated in the mouse experimental design and coordination. NK conceived of the study and participated in the rice experimental design and coordination. All authors read and approved the final manuscript.

## Supplementary Material

Additional file 1 **Additional Information**. It provides supplementary information about the detailed explanation of data and complicated mathematical derivations. Click here for file

## References

[B1] Lockhart DJ, Dong H, Byrne MC, Follettie MT, Gallo MV, Chee MS, Mittmann M, Wang C, Kobayashi M, Horton H (1996). Expression monitoring by hybridization to high-density oligonucleotide arrays. Nat Biotechnol.

[B2] Kidgell C, Volkman SK, Daily J, Borevitz JO, Plouffe D, Zhou Y, Johnson JR, Le Roch K, Sarr O, Ndir O (2006). A systematic map of genetic variation in *Plasmodium falciparum*. PLoS Path.

[B3] Winzeler EA, Richards DR, Conway AR, Goldstein AL, Kalman S, McCullough MJ, McCusker JH, Stevens DA, Wodicka L, Lockhart DJ, Davis RW (1998). Direct allelic variation scanning of the yeast genome. Science.

[B4] Turner TL, Hahn MW, Nuzhdin SV (2005). Genomic islands of speciation in *Anopheles gambiae*. PLoS Biol.

[B5] Borevitz JO, Liang D, Plouffe D, Chang HS, Zhu T, Weigel D, Berry CC, Winzeler E, Chory J (2003). Large-scale identification of single-feature polymorphisms in complex genomes. Genome Res.

[B6] Borevitz JO, Hazen SP, Michael TP, Morris GP, Baxter IR, Hu TT, Chen H, Werner JD, Nordborg M, Salt DE (2007). Genome-wide patterns of single-feature polymorphism in *Arabidopsis thaliana*. Proc Natl Acad Sci USA.

[B7] Kumar R, Qiu J, Joshi T, Valliyodan B, Xu D, Nguyen HT (2007). Single feature polymorphism discovery in rice. PLoS ONE.

[B8] Ronald J, Akey JM, Whittle J, Smith EN, Yvert G, Kruglyak L (2005). Simultaneous genotyping, gene-expression measurement, and detection of allele-specific expression with oligonucleotide arrays. Genome Res.

[B9] West MA, van Leeuwen H, Kozik A, Kliebenstein DJ, Doerge RW, St Clair DA, Michelmore RW (2006). High-density haplotyping with microarray-based expression and single feature polymorphism markers in *Arabidopsis*. Genome Res.

[B10] Rostoks N, Borevitz JO, Hedley PE, Russell J, Mudie S, Morris J, Cardle L, Marshall DF, Waugh R (2005). Single-feature polymorphism discovery in the barley transcriptome. Genome Biol.

[B11] Cui X, Xu J, Asghar R, Condamine P, Svensson JT, Wanamaker S, Stein N, Roose M, Close TJ (2005). Detecting single-feature polymorphisms using oligonucleotide arrays and robustified projection pursuit. Bioinformatics.

[B12] Luo ZW, Potokina E, Druka A, Wise R, Waugh R, Kearsey MJ (2007). SFP Genotyping from Affymetrix arrays is robust but largely detects *cis*-acting expression regulators. Genetics.

[B13] Kirst M, Caldo R, Casati P, Tanimoto G, Walbot V, Wise RP, Buckler ES (2006). Genetic diversity contribution to errors in short oligonucleotide microarray analysis. Plant Biotechnol J.

[B14] Greenhall JA, Zapala MA, Caceres M, Libiger O, Barlow C, Schork NJ, Lockhart DJ (2007). Detecting genetic variation in microarray expression data. Genome Res.

[B15] Hsieh WP, Chu TM, Wolfinger RD, Gibson G (2003). Mixed-model reanalysis of primate data suggests tissue and species biases in oligonucleotide-based gene expression profiles. Genetics.

[B16] Alberts R, Terpstra P, Li Y, Breitling R, Nap JP, Jansen RC (2007). Sequence polymorphisms cause many false *cis *eQTLs. PLoS ONE.

[B17] Gore M, Bradbury P, Hogers R, Kirst M, Verstege E, van Oeveren J, Peleman J, Buckler E, van Eijk M (2007). Evaluation of Target Preparation Methods for Single-Feature Polymorphism Detection in Large Complex Plant Genomes. Crop Science.

[B18] Bhat PR, Lukaszewski A, Cui X, Xu J, Svensson JT, Wanamaker S, Waines JG, Close TJ (2007). Mapping translocation breakpoints using a wheat microarray. Nucleic Acids Res.

[B19] Tusher VG, Tibshirani R, Chu G (2001). Significance analysis of microarrays applied to the ionizing radiation response. Proc Natl Acad Sci USA.

[B20] Windham MP (1995). Robustifying model fitting. J Roy Statist Soc Ser B.

[B21] Basu A, Harris IR, Hjort NL, Jones MC (1998). Robust and efficient estimation by minimising a density power divergence. Biometrika.

[B22] Jones MC, Hjort NL, Harris IR, Basu A (2001). A comparison of related density-based minimum divergence estimators. Biometrika.

[B23] Fujisawa H, Eguchi S (2008). Robust parameter estimation with a small bias against heavy contamination. J Multivariate Anal.

[B24] Scott DW (2001). Parametric statistical modeling by minimum integrated square error. Technometrics.

[B25] International Rice Genome Sequencing Project (2005). The map-based sequence of the rice genome. Nature.

[B26] Yu J, Wang J, Lin W, Li S, Li H, Zhou J, Ni P, Dong W, Hu S, Zeng C (2005). The genomes of *Oryza sativa*: A history of duplications. PLoS Biol.

[B27] Mouse Genome Sequencing Consortium (2002). Initial sequencing and comparative analysis of the mouse genome. Nature.

[B28] Abe K, Noguchi H, Tagawa K, Yuzuriha M, Toyoda A, Kojima T, Ezawa K, Saitou N, Hattori M, Sakaki Y (2004). Contribution of Asian mouse subspecies *Mus musculus molossinus *to genomic constitution of strain C57BL/6J, as defined by BAC-end sequence-SNP analysis. Genome Res.

[B29] Hampel FR, Ronchetti EM, Rousseeuw PJ, Stahel WA (1986). Robust statistics: The approach based on influence functions.

[B30] Marioni JC, Mason CE, Mane SM, Stephens M, Gilad Y (2008). RNA-seq: an assessment of technical reproducibility and comparison with gene expression arrays. Genome Res.

[B31] Cloonan N, Forrest AR, Kolle G, Gardiner BB, Faulkner GJ, Brown MK, Taylor DF, Steptoe AL, Wani S, Bethel G (2008). Stem cell transcriptome profiling via massive-scale mRNA sequencing. Nat Meth.

