# Supplementary material for: SNEP: Simultaneous detection of nucleotide and expression polymorphisms using Affymetrix GeneChip
Source: BMC Bioinformatics. 2009 May 6;10:131. doi: 10.1186/1471-2105-10-131 (PMC2706822; doi:10.1186/1471-2105-10-131)
Supplement: Additional file 1 — Additional Information. It provides supplementary information about the detailed explanation of data and complicated mathematical derivations. [file 1471-2105-10-131-S1.pdf]

Additional Information

Appendix

A1 Numerical algorithm to obtain the estimate

The robust parameter estimate can be obtained by the following iterative algorithm:

$$\begin{aligned}\lambda^{(a+1)} &= \sum_{j=1}^{J} z_j\, \phi(z_j;\boldsymbol{\theta}^{(a)})^{\gamma} \Big/ \sum_{j=1}^{J} \phi(z_j;\boldsymbol{\theta}^{(a)})^{\gamma} \\ (\kappa^2)^{(a+1)} &= \left\{ \sum_{j=1}^{J} z_j^2\, \phi(z_j;\boldsymbol{\theta}^{(a)})^{\gamma} \Big/ \sum_{j=1}^{J} \phi(z_j;\theta^{(a)})^{\gamma} - \left(\lambda^{(a+1)}\right)^2 \right\} (1+\gamma).\end{aligned}$$

For details, see Windham (1995) and Fujisawa and Eguchi (2008). SNEP adopts the median and MAD (median absolute deviation) as the initial values for the mean and variance parameters.

A2 Asymptotic property of $\hat{\boldsymbol{\theta}}$

The estimationg equation of $\hat{\boldsymbol{\theta}}$ can be expressed by

$$\frac{1}{J}\sum_{j=1}^{J} \boldsymbol{h}(z_j;\boldsymbol{\theta}) = 0,$$

where $l(z;\boldsymbol{\theta}) = \log\phi(z;\boldsymbol{\theta})$, $\boldsymbol{s}(z;\boldsymbol{\theta}) = \partial l/\partial\boldsymbol{\theta}$, and

$$\boldsymbol{h}(z_j;\boldsymbol{\theta}) = \phi(z_j;\boldsymbol{\theta})^{\gamma}\boldsymbol{s}(z_j;\boldsymbol{\theta}) \int \phi(z;\boldsymbol{\theta})^{1+\gamma} dz - \phi(z_j;\boldsymbol{\theta})^{\gamma} \int \phi(z;\boldsymbol{\theta})^{1+\gamma}\boldsymbol{s}(z;\boldsymbol{\theta}) dz.$$

This type of estimation is called M-estimation. Let $\boldsymbol{\theta}^{\dagger}$ be the minimizer of (2.1) on population level. Let $g(z)$ be the data generating density function. From the theory of the M-estimator, it holds that

$$\sqrt{J}\left(\hat{\boldsymbol{\theta}} - \boldsymbol{\theta}^{\dagger}\right) \xrightarrow{d} N\left(0, \Sigma_g(\boldsymbol{\theta}^{\dagger})\right),$$

where $\Sigma_g(\boldsymbol{\theta}) = J_g(\boldsymbol{\theta})^{-1} I_g(\boldsymbol{\theta}) J_g(\boldsymbol{\theta})'^{-1}$,

$$J_g(\boldsymbol{\theta}) = \mathrm{E}_g\left[\frac{\partial}{\partial\boldsymbol{\theta}'}\boldsymbol{h}(z;\boldsymbol{\theta})\right], \qquad I_g(\boldsymbol{\theta}) = \mathrm{E}_g\left[\boldsymbol{h}(z;\boldsymbol{\theta})\boldsymbol{h}(z;\boldsymbol{\theta})'\right].$$

Suppose that $g(z) = (1-\varepsilon)\phi(z;\boldsymbol{\theta}^*) + \varepsilon\delta(z)$, where $\delta(z)$ is the contamination density related to outlier and ε is the ratio of outlier. Assume some conditions related to the situation where $\delta(z)$ lies on the tail of $\phi(z;\boldsymbol{\theta}^*)$. It then follows that $\boldsymbol{\theta}^{\dagger} \approx \boldsymbol{\theta}^*$ and $\Sigma_g(\boldsymbol{\theta}^{\dagger}) \approx \Sigma_f(\boldsymbol{\theta}^*)/(1-\varepsilon)$. For detailed conditions and derivations, see Fujisawa and Eguchi (2008).

A3 Detailed form of $\hat{\boldsymbol{\kappa}}_j$

We can consider that $\hat{\lambda}$ and $\hat{\lambda}_j$ are approximately independent when J is sufficiently large. Hence, we can approximate the variance of $\hat{\lambda}-\hat{\lambda}_j$ by $\kappa_\lambda^2+\mathrm{Var}[z_j]=\kappa_\lambda^2+2\kappa^2/k$. This can be estimated using the estimate of κ_λ^2, suggested in the next section, and $\hat{\kappa}^2$.

A4 Detailed form of $\hat{\boldsymbol{\kappa}}_{\boldsymbol{\lambda}}$

Let κ_λ^2 be the $(1,1)$-component of $\Sigma_g(\boldsymbol{\theta}^\dagger)/J$. By using the approximation relation $\Sigma_g(\boldsymbol{\theta}^\dagger)\approx\Sigma_f(\boldsymbol{\theta}^*)/(1-\varepsilon)$, it is shown after simple but troublesome calculation that

$$\kappa_\lambda^2\approx\frac{1}{1-\varepsilon}\frac{(1+\gamma)^2c_\gamma^2(\kappa^2)}{(1+2\gamma)c_{2\gamma}(\kappa^2)}\frac{2}{K}\kappa^2\frac{1}{J},$$

where $c_\gamma(\kappa^2)=\left\{\int\phi(z;\lambda,\kappa^2)^{1+\gamma}dz\right\}^{-1}=(2\pi)^{\gamma/2}(1+\gamma)^{1/2}(\kappa^2)^{\gamma/2}$. Let

$$T_\gamma=\frac{1}{n}\sum_{j=1}^{J}(z_j-\hat{\lambda})^2\phi(z_j;\hat{\boldsymbol{\theta}})^\gamma.$$

By using a similar way to the above, we can easily see that the convergence limit of T_γ is approximated by

$$(1-\varepsilon)\frac{1}{(1+\gamma)c_\gamma(\kappa^2)}\frac{2}{K}\kappa^2.$$

Consequently, we can propose an appropriate estimate $\hat{\kappa}_\lambda^2$ of κ_λ^2 by $(T_{2\gamma}/T_\gamma^2)(2/K)^2\hat{\kappa}^4/J$.

References

Fujisawa, H. and Eguchi, S. (2008). Robust parameter estimation with a small bias against heavy contamination. *J. Multivariate Anal.*, **99**, 2053–2081.

Windham, M.P. (1995). Robustifying model fitting. *J. Roy. Statist. Soc. Ser. B*, **57**, 599–609.

mRNA data

RNA isolation and array experiment

The Affymetrix GeneChip Rice Genome Array were designed for targeting near the 3' region of 48,564 *japonica* transcripts and 1,260 *indica* transcripts based on the UniGene Build #52 (May 7, 2004), GenBank mRNAs (July 13, 2004), and 59,712 predicted genes from TIGR's osa1 version 2.0 (http://www.affymetrix.com/products/arrays/specific/rice.affx). It consisted of 57,381 probe sets containing 631,066 probes. Signal intensities for the two fully sequenced rice cultivars, *japonica* rice "Nipponbare" (International Rice Genome Sequencing Project, 2005) and *indica* one "93-11" (Yu *et al.*, 2005), were observed by hybridizing their mRNA to rice arrays.

The Affymetrix GeneChip Mouse Genome 430 2.0 Arrays were designed for targeting $\geq$39,000 transcripts based on the UniGene Build #107 (June, 2002) by genome sequence of a laboratory inbred strain, C57BL/6J (referred to below as B6), whose genome is predominantly derived from West European subspecies *Mus musculus domestius* (*M. m. domesticus*). It consisted of 45,101 probe sets containing 496,468 probes. Signal intensities for the two inbred strains B6 and MSM/Ms (*Mus musculus molossinus*) were observed by hybridizing their mRNA to mouse arrays. MSM/Ms is derived from the Japanese wild mouse *M. m. molossinus* (Moriwaki, 1994).

Total RNA was extracted from 2cm young panicles of both Nipponbare and 93-11 using a QIAGEN RNeasy Plant Mini Kit as per the manufacturer's protocol (QIAGEN GmbH, Hilden, Germany). Labeled cRNA was prepared and hybridized to Affymetrix GeneChip Rice Genome Arrays according to the manufacturer's guidelines (Affymetrix, Santa Clara, CA). The GeneChips were scanned with an Affymetrix GeneArray 3000 scanner and the raw .CEL files were generated by the Affymetrix GeneChip Operating Software (GCOS) version 1.3. Five biological replicates were obtained for each condition. For mouce, total RNA was extracted from livers of B6 and MSM/Ms adult mice using a QIAGEN RNeasy Mini Kit as per the manufacturer's protocol. RNA labeling and hybridization to Affymetrix GeneChip Mouse Genome 430 2.0 Arrays and data generation were done using the same method as rice. Two biological replicates were obtained for the mouse data. All microarray data from this work are available from the Center for Information Biology gene EXpression (CIBEX) database (http://cibex.nig.ac.jp/index.jsp) under accession numbers CBX50 and CBX54.

For a standard analysis of mRNA data, the expresso in the affy package (Gautier *et al.*, 2004) with Bioconductor software (http://www.bioconductor.org/) was used as MAS 5.0 and each expression level was estimated using the one-step Tukey's bi-weight algorithm without any background correction or normalization.

Sequence Analysis

For all 631,066 probes on the rice GeneChips, possible target regions in the Nipponbare genome (total 371Mb from GenBank/Embl/DDBA accession:AP008207 to AP008218; International Rice Genome Sequencing Project, 2005) and the 93-11 genome (total 479Mb, including the unmapped contigs (105Mb) from version 2003-08-01 BGI; Yu *et al.*, 2005) were searched by 'blastn' version 2.2.8 (Altschul *et al.*, 1997) with the following conditions; the expectation value was 20, the match score was 1, the mismatch score was -3, the cost to a gap open was 5, and the cost of extension to a gap was 2. A perfect match region with the probe target sequence was scored 25. A single mismatch between the genome sequence and a probe target scored from 24 to 21, depending on the mismatch position. When the mismatch was in the distal three bases, 'blastn' counted a continuous match, however, if it was in an inner position, the score was 21. Similarly, a single insertion in a genome sequence scored from 24 to 18. When the alignment length of the 'blastn' output was shorter than a probe length, a deficient sequence was retrieved from the genome sequence and then the matched bases were counted. If a probe sequence hit singly in a certain region, with more than 23 match bases on genome sequence by the above search, we regarded the probe to be singly copy to the genome. SNPs in the probe target regions between B6 and MSM/Ms were retrieved from the NIG mouse genome database (http://molossinus.lab.nig.ac.jp/msmdb/).

References

Altschul, S. *et al.* (1997). Gapped BLAST and PSI-BLAST: a new generation of protein database search programs. *Nucl. Acids Res.*, **25**, 3389–3402.

Gautier, L. *et al.* (2004). Affy–analysis of Affymetrix GeneChip data at the probe level. *Bioinformatics*, **20**, 307–315.

International Rice Genome Sequencing Project (2005). The map-based sequence of the rice genome. *Nature*, **436**, 793–800.

Moriwaki, K. (1994). Wild mouse from geneticist's viewpoint. In Moriwaki,K. *et al.* (eds.), *Genetics in wild mice: Its application to biomedical research.* Japan Scientific Press/Karger, Tokyo, pp. xiii-xxiv.

Yu, J. *et al.* (2005). The genomes of *Oryza sativa*: A history of duplications. *PLoS Biol.*, **3**, e38.
